# Comparison of Techniques for Respiratory Rate Extraction from Electrocardiogram and Photoplethysmogram

**DOI:** 10.3390/s25165136

**Published:** 2025-08-19

**Authors:** Alfonso Maria Ponsiglione, Michela Russo, Maria Giovanna Petrellese, Annalisa Letizia, Vincenza Tufano, Carlo Ricciardi, Annarita Tedesco, Francesco Amato, Maria Romano

**Affiliations:** 1 Department of Electrical Engineering and Information Technology, University of Naples Federico II, Via Claudio 22, 80125 Naples, Italy; carlo.ricciardi@unina.it (C.R.); framato@unina.it (F.A.); mariarom@unina.it (M.R.); 2 Neurological Clinic of Hospital San Giovanni di Dio and Ruggi d’Aragona, Via San Leonardo, 80132 Salerno, Italy; 3 Teoresi S.p.A. Group, Via Perugia 24, 10152 Torino, Italy; 4 Department of Public Healtcare, University of Naples Federico II, Via S. Pansini, 80131 Napoli, Italy; annarita.tedesco@unina.it

**Keywords:** respiratory rate, ECG-derived respiration, PPG-derived respiration, signal processing, features extraction

## Abstract

Background: Respiratory rate (RR) is a key vital sign and one of the most sensitive indicators of physiological conditions, playing a crucial role in the early identification of clinical deterioration. The monitoring of RR using electrocardiography (ECG) and photoplethysmography (PPG) aims to overcome limitations of traditional methods in clinical settings. Methods: The proposed approach extracts RR from ECG and PPG signals using different morphological and temporal features from publicly available datasets (iAMwell and Capnobase). The algorithm was used to develop and test with a selection of relevant ECG (e.g., R-peak, QRS area, and QRS slope) and PPG (amplitude and frequency modulation) characteristics. Results: The results show promising performance, with the ECG-derived signal using the R-peak–based method yielding the lowest error, with a mean absolute error of 0.99 breaths/min in the iAMwell dataset and 3.07 breaths/min in the Capnobase dataset. In comparison, the RR PPG-derived signal showed higher errors of 5.10 breaths/min in the iAMwell dataset and 10.66 breaths/min in the Capnobase dataset, for the FM and AM method, respectively. Bland–Altman analysis revealed a small negative bias, approximately −0.97 breaths/min for the iAMwell dataset (with limits of agreement from −2.62 to 0.95) and −1.16 breaths/min for the Capnobase dataset (limits of agreement from −3.37 to 1.10) in the intra-subject analysis. In the inter-subject analysis, the bias was −0.84 breaths/min (limits of agreement from −1.76 to 0.20) for iAMwell and −1.22 breaths/min (limits of agreement from −7.91 to 5.35) for Capnobase, indicating a slight underestimation. Conversely, the PPG-derived signal tended to overestimate RR, resulting in higher variability and reduced accuracy. These findings highlight the higher reliability of ECG-derived features for RR estimation in the analyzed datasets. Conclusion: This study suggests that the proposed approach could guide the design of cost-effective, non-invasive methods for continuous respiration monitoring, offering a reliable tool for detecting conditions like stress, anxiety, and sleep disorders.

## 1. Introduction

The respiratory rate (RR) is one of the most informative indicators of physiological state and a vital sign for the early detection of clinical deterioration. Continuous monitoring of respiration plays a crucial role in the diagnosis and management of a wide range of conditions. In healthy subjects at rest, normal RR ranges between 16 and 20 breaths per minute; an increased RR may indicate fever, dehydration, anxiety, mental stress, asthma, pulmonary diseases, or heart conditions [1,2].

Conversely, a decreased RR can be associated with drug use, metabolic abnormalities, sleep apnea events, and other disorders [3]. In addition, the coupling between respiration and heart rate represents a key parameter both for the detection of pathological conditions [4,5,6,7] and for studying cardiorespiratory system interactions [8].

In general hospital settings, respiration can be measured using both direct and indirect methods. Direct measurements involve assessing air flow, pressure, and temperature through the lungs using devices such as spirometers. Indirect measurements rely on thoracic volume changes, detected through transthoracic inductance or impedance plethysmography systems [9].

However, these methods present several limitations—they are often time-consuming, costly, and require bulky equipment. Furthermore, contact-based sensors or masks may interfere with natural breathing patterns. The extraction of RR is also highly susceptible to motion artifacts, making these systems less suitable for applications that require continuous, unobtrusive monitoring, such as in polysomnography or stress assessment.

For these reasons, there is a clinical need for algorithms capable of extracting the RR from waveforms that are already collected in the hospital setting. One such approach involves estimating RR from electrocardiogram (ECG) and photoplethysmography (PPG) signals [10,11,12,13,14]. Indeed, respiratory activity influences both ECG and PPG signals. In the case of the ECG signal, respiration affects heart rate variability as well as beat morphology. On the one hand, respiration modulates heart rate, which increases during inspiration and decreases during expiration, a phenomenon known as respiratory sinus arrhythmia (RSA) [15,16]. On the other hand, ECG morphology is also influenced by respiration due to relative movements of the electrodes with respect to the heart and changes in thoracic impedance caused by lung filling and emptying [17]. As for PPG signals, the reduction in stroke volume during ventricular filling induces amplitude variations in the PPG signal, reflecting respiratory activity [18].

Ultimately, both ECG and PPG signals are influenced by respiratory activity through three types of modulation—amplitude modulation (AM), baseline wander (BW), and frequency modulation (FM) [19]. AM refers to variations in peak amplitude observed in both ECG and PPG signals. BW affects the low-frequency baseline of these signals, introducing slow fluctuations unrelated to physiological activity [20,21,22]. Finally, FM appears as beat-to-beat variations in the duration of cardiac cycles, detectable in both ECG and PPG [19,23]. BW and AM in the ECG are also influenced by changes in the orientation of the heart’s electrical axis relative to the electrodes, as well as variations in thoracic impedance [19]. BW in the PPG signal is caused by changes in tissue blood volume [21], while AM in the PPG is associated with respiration-induced changes in stroke volume [22]. FM in ECG and PPG is due to RSA, which causes the heart rate to increase during inspiration and decrease during exhalation [19]. The intensity of each modulation can vary between subjects, depending on their physiological parameters and other characteristics such as gender, age, health condition, weight, and height [19,24].

Therefore, while ECG measures heart rhythm and electrical activity, and PPG is used for detecting blood volume changes in the microvascular bed via pulse oximetry, both signals are closely correlated with the RR and are routinely acquired in clinical settings. This makes them strong candidates for inexpensive and non-invasive RR monitoring. Over the past decade, Charlton et al. [25] conducted a comprehensive comparison of over 100 algorithms for estimating RR from ECG and PPG signals. Their review covered a wide range of methods that use either ECG or PPG as input signals [26,27,28,29,30]. Most of these algorithms focus on optimizing the filtering stages to enhance the signal-to-noise ratio, thereby improving the reconstruction of the respiratory signal [31,32,33].

In contrast, the present study introduces a methodological pipeline designed to guide researchers in extracting RR from ECG or PPG signals while avoiding unnecessary processing steps. To ensure reproducibility and promote an adaptable workflow, we tested the proposed pipeline on two publicly available datasets: iAMwell [34] and Capnobase [35]. Specifically, we analyzed a wide range of morphological and temporal features derived from ECG signals (R-peak, QRS area, up-slope, down-slope) and PPG signals (frequency modulation and amplitude modulation) to identify and compare the most accurate and robust features for RR estimation, accounting for both intra- and inter-subject variability. The results provide valuable insights that can inform the development of efficient, non-invasive, and low-cost systems for continuous respiratory monitoring.

## 2. Materials and Methods

### 2.1. Dataset

We evaluated the proposed method on two publicly available datasets, the iAMwell open-source dataset [34] and Capnobase [35], to verify the generalization performance of the algorithm across different scenarios.

The iAMwell dataset contains simultaneously recorded ECG, PPG, and respiratory signals from athletes and non-athletes during an experimental protocol, including rest, exercise, and recovery phases. All signals were sampled at 2000 Hz. In this study, we focused on the acceleration phase, lasting approximately 8 min, characterized by a gradual increase in running speed and corresponding physiological changes.

The Capnobase dataset includes simultaneously recorded ECG, PPG, and respiratory signals from 42 subjects undergoing elective surgery and routine anesthesia. The recordings, lasting about 8 min, were sampled at 300 Hz. We considered only 19 spontaneous breathing cases for our analysis.

### 2.2. Signal Processing and Analysis

Before detailing the algorithms for ECG and PPG analysis, it is important to highlight that all records, including ECG, PPG, and respiratory signals, were divided into segments. To determine the optimal segment length for accurate RR prediction, we tested windows of 20, 30, and 60 s to allow a fair comparison. The 30-s window consistently provided the best accuracy in RR estimation, in agreement with previous literature, which demonstrated that longer windows tend to result in higher mean errors [36,37].

#### 2.2.1. ECG Pre-Processing and Analysis

**Figure 1:**
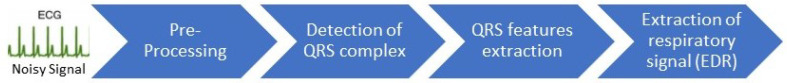
ECG processing workflow.

The following flowchart (Figure 1) illustrates the ECG analysis step-by-step.

As is commonly done to improve the signal-to-noise ratio (SNR) and ensure accurate detection of the QRS complex while enhancing the quality of the extracted respiratory signal, denoising of the raw ECG signal was applied. Each step of the algorithm, shown in the workflow of Figure 1, follows traditional signal processing methodologies widely discussed in the literature [38,39,40]. The selection of parameters for each processing step was made empirically, based on the characteristics of the ECG signal and the specific requirements of the analysis.

**Figure 2:**
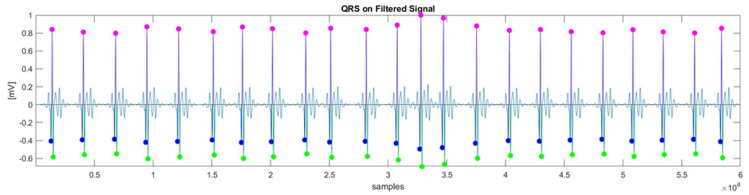
QRS complex detection algorithm output. The colored points are referred to as Q-peak (blue), R-peak (pink), and S-peak (green), respectively.

In the preprocessing stage, a cascade of low-pass and high-pass filters was used to remove the P and T waves, as well as electromyography artifacts. The cut-off frequencies for the band-pass filter were set to 5 Hz (low-pass) and 40 Hz (high-pass). The next step, QRS complex detection, can be challenging due to physiological variability and various types of noise present in the ECG signal. The Pan–Tompkins algorithm [41], known for its robustness in detecting QRS complexes, was employed. The Q and S waves were identified by searching for local minima before and after the R-peak, as illustrated in Figure 2.

**Figure 3:**
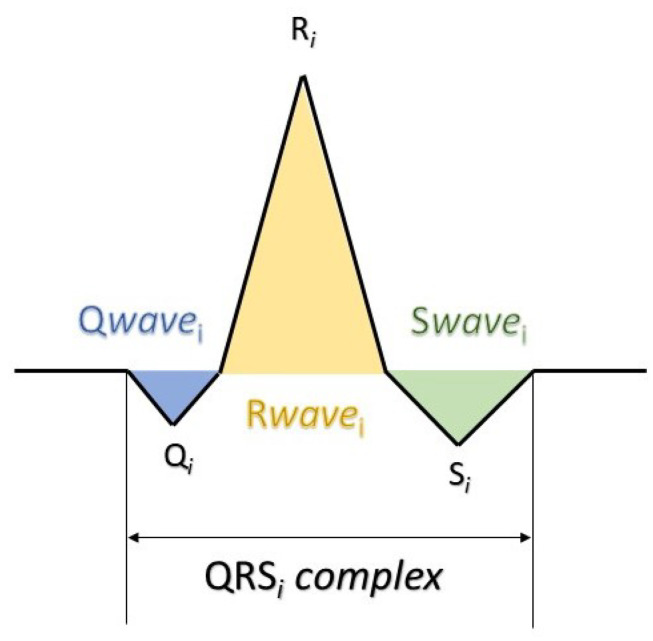
QRS complex detection algorithm output. The colored points are referred to as Q-peak (blue), R-peak (pink), and S-peak (green), respectively.

For QRS features extraction, four morphological features were extracted: R-peak (amplitude), QRS slopes (up-slope and down-slope), and QRS area, as described in [42]. Let us denote by $QRS_{i}$ the *i*-th QRS complex of the total ECG signal (Figure 2); the computation of the morphological features is outlined as follows (Figure 3):

(1) $$R_{i}=max\left(x_{j}\right){|}_{j\in \left[QRS_{i}\right]}$$

(2) $$UpSlope_{i}=max\left(abs\left(x_{j}-x_{j-1}\right)\right){|}_{j\in \left[Q_{i},R_{i}\right]}$$

(3) $$DownSlope_{i}=max\left(abs\left(x_{j+1}-x_{j}\right)\right){|}_{j\in \left[R_{i},S_{i}\right]}$$

The area is calculated by approximating the integral with trapezoids. The following equation describes the total area used in this study:

(4) $$Area\left(QRS_{i}\right)=abs\left(area\left(Qwave_{i}\right)\right)+abs\left(area\left(Rwave_{i}\right)\right)+abs\left(area\left(Swave_{i}\right)\right)$$

Finally, based on these features, the ECG-derived respiration (EDR) signals were extracted. To reconstruct a continuous respiration waveform, cubic spline interpolation was applied to the detected peaks of these features. Since different QRS parameters are influenced by respiration in varying ways, a separate EDR signal was generated for each extracted QRS feature, enabling a more comprehensive representation of the respiratory pattern.

#### 2.2.2. PPG Pre-Processing and Analysis

**Figure 4:**
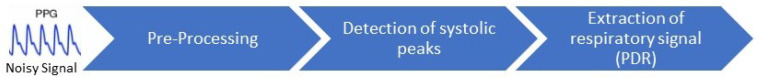
Photoplethysmogram processing workflow.

Figure 4 illustrates the step-by-step processing of the PPG signal. Similar to the ECG processing, the methodologies for PPG signal processing were based on established techniques from the literature, and the parameters were set empirically.

The raw PPG signal was first filtered using an 8th-order Butterworth low-pass filter with a cut-off frequency of 10 Hz to remove high-frequency noise. Next, an 11-level wavelet decomposition was applied to further smooth the signal, as recommended in the literature for PPG preprocessing [43,44,45,46]. To enhance the extraction of respiratory-related variations, a Savitzky–Golay polynomial filter was employed [47] for more precise detection of RR from the reference signals.

**Figure 5:**
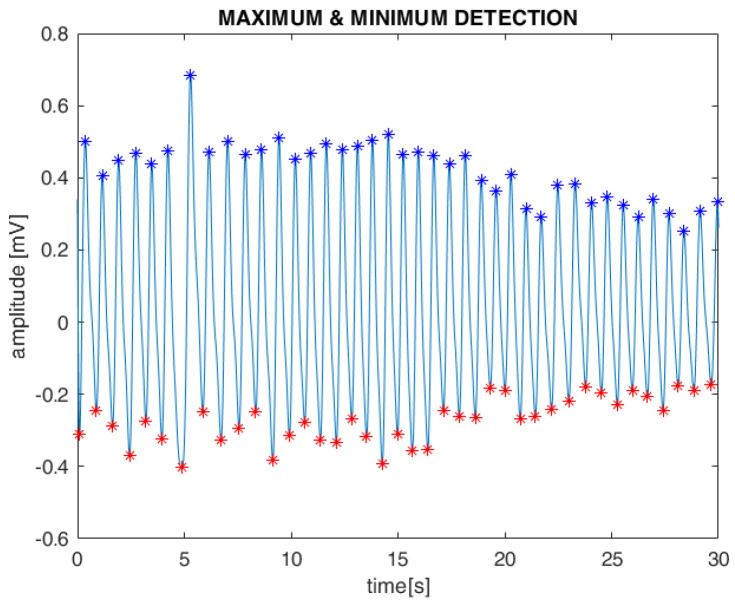
PPG peaks detection: colored points indicate maximum values (blue) and minimum values (red), respectively.

Subsequently, the systolic maximum and minimum values of the PPG waveform, along with the intervals between them, were detected using standard MATLAB toolboxes. These features were extracted from both the AM and FM components of the filtered PPG signals (Figure 5). Finally, as with the ECG, cubic spline interpolation was used to reconstruct the PPG-derived respiration (PDR) signal.

### 2.3. Respiratory Rate Extraction

In each case, RR was estimated by counting the number of respiratory peaks within a fixed 30-s time window. RR was computed for each reference respiratory signal and derived from the individual ECG and PPG characteristics, using the following formula:

(5) $$\mathrm{RR}=\frac{N_{\mathrm{peaks}}}{T}\cdot 60$$

where $N_{\mathrm{peaks}}$ is the number of detected respiratory peaks in a window of duration T=30 s. RR is expressed in breaths per minute (breaths/min).

### 2.4. Quantitative Analysis

A quantitative analysis was carried out using the following three performance metrics:

- Mean Absolute Error (MAE)
  (6) $$\mathrm{MAE}=\frac{1}{N}\sum_{i=1}^{N}\left|f_{i}-\hat{f_{i}}\right|$$
- Mean Absolute Percentage Error (MAPE)
  (7) $$\mathrm{MAPE}=\frac{100}{N}\sum_{i=1}^{N}\frac{f_{i}-\hat{f_{i}}}{f_{i}}$$
- Root Mean Square Error (RMSE)
  (8) $$\mathrm{RMSE}=\sqrt{\frac{1}{N}\sum_{i=1}^{N}{\left(f_{i}-\hat{f_{i}}\right)}^{2}}$$

where $f_{i}$ and $\hat{f_{i}}$ represent the reference and estimated RRs, respectively, and *N* is the number of observations.

Additionally, the Pearson correlation coefficient (ρ) was computed to assess the linear relationship between derived and reference RR. Interpretation of ρ typically follows these conventional thresholds: values below 0.3 indicate a weak correlation, values between 0.3 and 0.7 suggest a moderate correlation, and values above 0.7 are considered indicative of a strong correlation. This statistical measure complements the error-based metrics by capturing the degree of trend agreement between signals.

The analysis was conducted on two levels, as follows: intra-subject analysis, where for each subject, the RR values were computed across all time windows (e.g., for subject 1, we considered all 15 windows), allowing us to assess how each subject’s respiratory rate varied over time; and inter-subject analysis, where for each time window, the RR values were compared across all subjects (e.g., for time window 1, we considered the RR of all subjects), enabling us to evaluate variability between subjects. This dual approach allowed us to capture both intra-subject variability over time and differences in estimation performance across subjects. The evaluation metrics (MAE, RMSE, MAPE, and ρ) were calculated for the overall values, obtained as the average of the intra-subject means followed by the average of the inter-subject means.

Furthermore, to assess and compare the accuracy and reliability of the two measurements, the Wilcoxon signed-rank test was performed on the within and between analysis by comparing the average derived and reference RRs.

Moreover, Bland–Altman (BA) analysis in and between subjects was used to visually assess the agreement between the two measurements. In the graphical representation of BA analysis, the mean of the two measurements is plotted on the x-axis, while the difference between the measurements is plotted on the y-axis. To obtain the limits of agreement, defined as the mean of the differences ±1.96 times the standard deviation (i.e., 95% of the confidence interval), the mean of differences (bias) and the standard deviation of differences were calculated. This method allows for evaluating errors, such as the distance of bias from zero. The overall statistical significance was set to α=0.05.

## 3. Results

**Figure 6:**
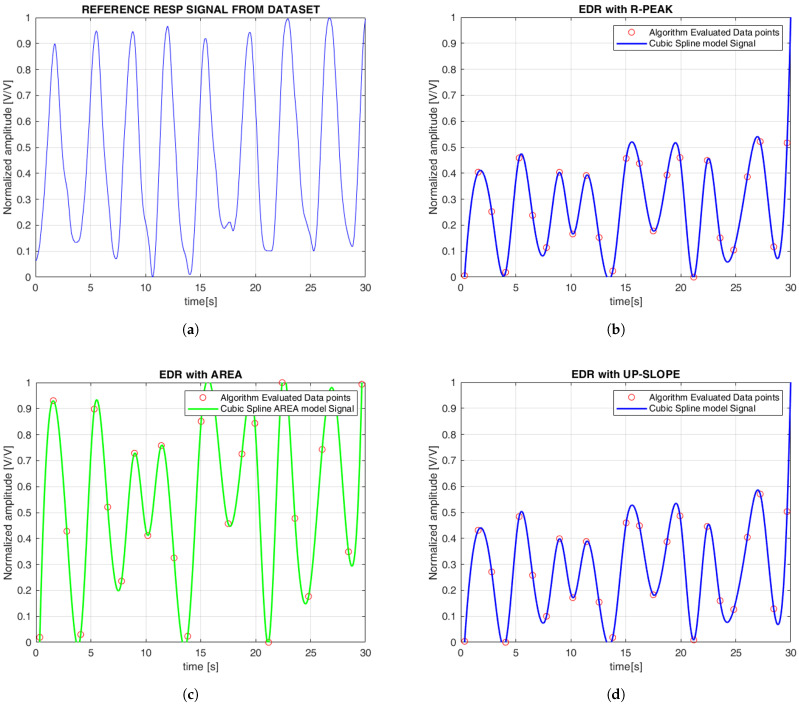

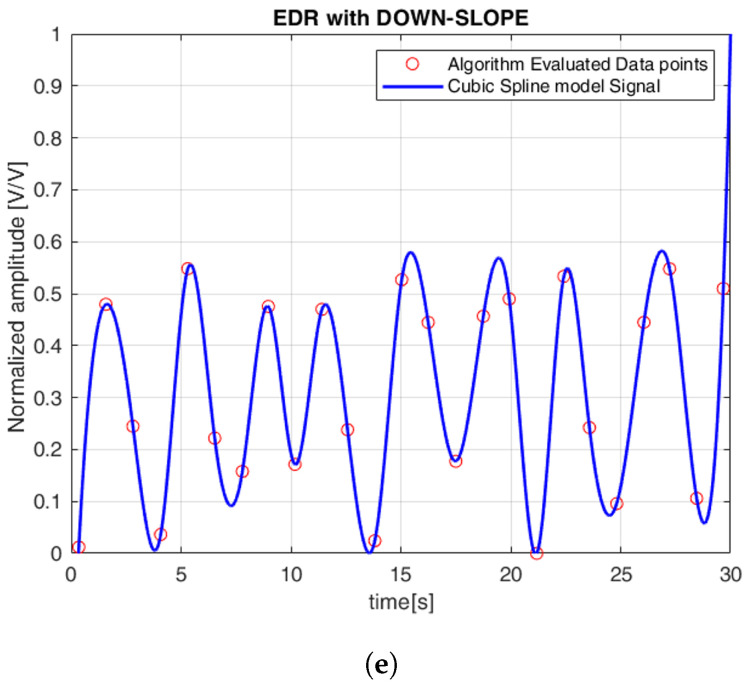
Comparison of respiratory signals derived from different methods: (**a**) reference respiratory signal; (**b**) electrocardiogram-derived respiration (EDR) derived from R-peak; (**c**) EDR derived from area; (**d**) EDR derived from up-slope; (**e**) EDR derived from down-slope. The red circles represent the discrete data points obtained from the applied methods over time. The continuous line represents the EDR signal reconstructed using cubic spline interpolation based on these points. The signal is normalized and expressed in arbitrary units [V/V], with time shown in seconds [s].

**Figure 7:**
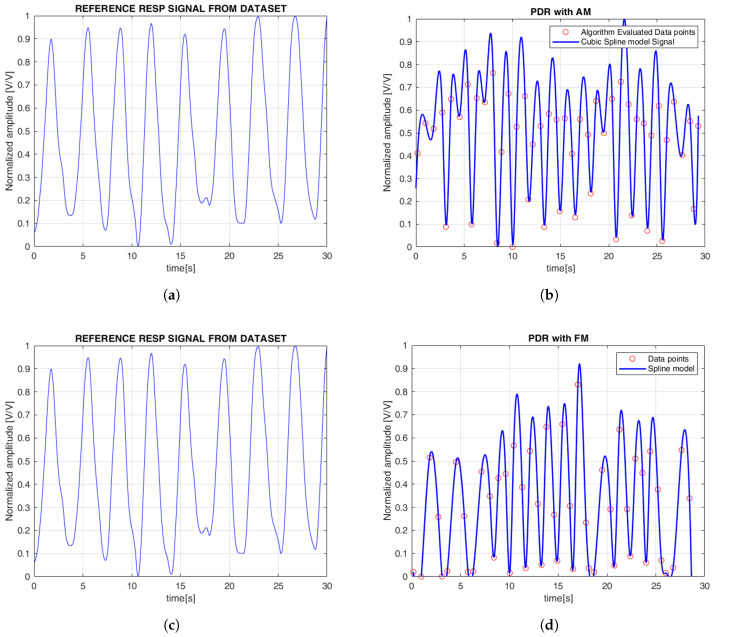
(**a**,**c**) Reference respiratory signals are displayed; (**b**) the photoplethysmogram-derived respiration (PDR) with amplitude modulation (AM); (**d**) PDR from frequency modulation (FM). The red circles represent the discrete data points obtained from the applied methods over time. The continuous line represents the PDR signal reconstructed using cubic spline interpolation based on these points. The signal is normalized and expressed in arbitrary units [V/V], with time shown in seconds [s].

In this section, we present both qualitative and quantitative evaluations of the ECG- and PPG-estimated signals compared to the reference signals. Figure 6 illustrates the continuous EDR signal reconstructed using cubic spline interpolation, applied to QRS morphological features. To simplify the comparison between the reference and estimated signals, the amplitudes were normalized, with all plots displayed between 0 and 1. Specifically, the R-peak, QRS slope (both up-slope and down-slope), and QRS area were extracted, with each feature generating a separate EDR signal that reflects the respiratory variations associated with that particular morphological feature. Similarly, Figure 7 shows the PDR signals derived from the AM and FM components of the PPG signal. Both features were analyzed, and cubic spline interpolation was used to reconstruct a continuous respiration signal derived from the PPG.

Table 1 shows the performance metrics for the ECG and PPG signals for both datasets. The results clearly show that ECG signals outperform PPG signals in terms of accuracy of breath rate estimation, as reflected in consistently lower MAE, MAPE, and RMSE values for ECG-based methods. Specifically, ECG-based methods (such as the R peak, QRS area, and QRS slope) showed lower and more consistent errors in both datasets. In the iAMwell dataset, MAE ranged from 0.99 to 1.04 breaths/min, while in the Capnobase dataset, it increased to between 3.07 and 3.74 breaths/min, still within a reasonably low error range. In contrast, PPG-based methods (FM and AM) showed significantly higher MAE values, ranging between 5.10 and 5.12 breaths/min in iAMwell and increasing substantially in Capnobase (10.66 and 13.90 breaths/min), indicating poorer performance. Looking at MAPE, the ECG methods maintained relatively low errors (9.45–9.94% in iAMwell and around 30.78% in Capnobase), while PPG showed substantially higher error rates: 26.65–27.17% in iAMwell and more than 100% in Capnobase, reflecting a much less reliable estimation. The RMSE values follow the same trend—the ECG resulted in 2.68–2.86 pbm in iAMwell and 11.71–13.46 breaths/min in Capnobase, while the PPG results were consistently worse in both datasets.

Regarding correlation, a moderate and statistically significant relationship was observed for ECG-based methods in both data sets. Specifically, for the R-peak–based method, the correlation coefficient was ρ=0.700 in the iAMwell dataset (*p*-value = 0.010) and ρ=0.642 in the Capnobase dataset (*p*-value = 0.015). For the QRS area method, the correlation was ρ=0.684 in iAMwell and ρ=0.535 in Capnobase (*p*-values = 0.021 and 0.016, respectively). The up-slope method yielded ρ=0.672 in iAMwell (*p*-value = 0.024), while the down-slope method showed a lower correlation of ρ=0.600 (*p*-value = 0.036). In Capnobase, the QRS slopes showed a significant correlation (*p*-value = 0.029, and *p*-value = 0.038, respectively), but with respect to ρ < 0.50. In contrast, PPG-based methods demonstrated weak correlations in both datasets, with all ρ values below 0.5, indicating a limited association between PPG-derived estimates and the reference respiratory rate.

Table 2 and Table 3 present the within-subject comparison between the derived and reference average RR values for both datasets, respectively.

Table 4 and Table 5 present the between-subject comparison between the derived and reference average RR values for each dataset, respectively.

To assess the agreement between the estimated and reference RR, the Bland–Altman bias and limits of agreement for each method, for both ECG and PPG signals, and for both datasets (iAMwell and Capnobase), are summarized in Table 6.

It is worth noting that ECG-based methods tend to underestimate the RR, whereas PPG-based methods generally overestimate it. Among all evaluated techniques, the R-peak–based method demonstrated the most stable and accurate performance, with consistently low bias values and performance metrics in both within-subject and between-subject analyses, and across the iAMwell and Capnobase datasets. Given its robustness, low systematic error, and consistent performance, the BA plots are reported only for the R-peak–based method, as a representative example of the best-performing approach.

**Figure 8:**
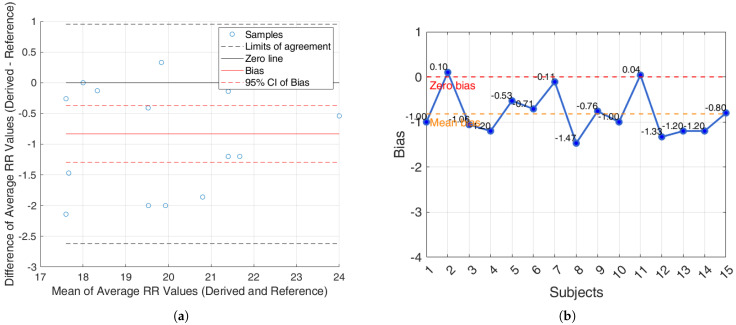
iAMwell dataset. (**a**) Bland–Altman plot for the R-peak based on intra-subject analysis, showing agreement between the average derived and reference RRs; (**b**) bias computed on each subject (breaths/min). The red line indicates the zero-bias reference, while the orange line represents the mean bias.

For the iAMwell dataset, the BA analysis based on the agreement within subjects is shown in Figure 8a. In Figure 8b, the bias for each subject is reported.

**Figure 9:**
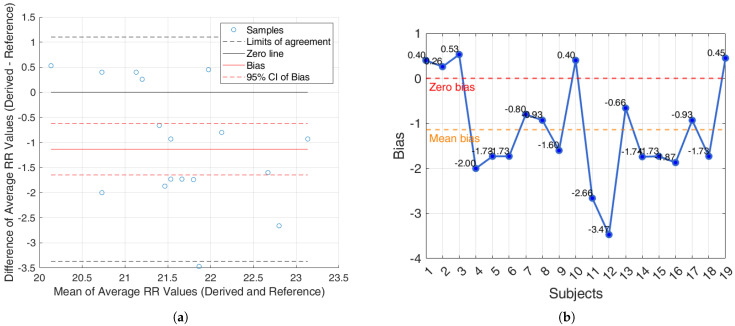
Capnobase dataset. (**a**) Bland–Altman plot for the R-peak based on intra-subject analysis, showing agreement between the average derived and reference RRs; (**b**) bias computed on each subject (breaths/min). The red line indicates the zero-bias reference, while the orange line represents the mean bias.

For the Capnobase dataset, the BA analysis based on the agreement within subjects is shown in Figure 9a. In Figure 9b, the bias for each subject is reported.

**Figure 10:**
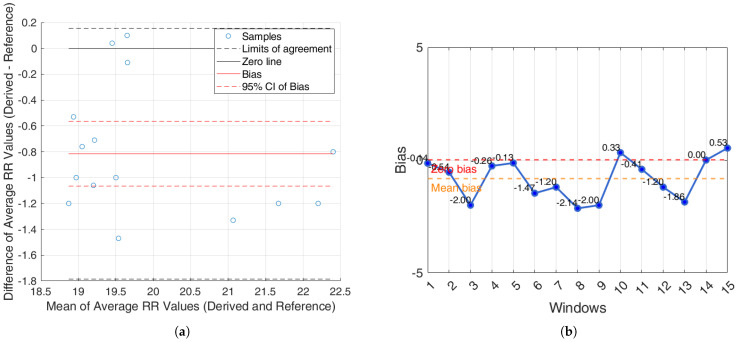
iAMwell dataset. (**a**) Bland–Altman plot for the R-peak based on inter-subject analysis, showing agreement between the average derived and reference RRs; (**b**) bias computed on each window (breaths/min). The red line indicates the zero-bias reference, while the orange line represents the mean bias.

For the iAMwell dataset, the BA analysis based on the agreement between subjects, which shows the average RR (derived and reference), is shown in Figure 10a. In Figure 10b, the bias for each window is reported.

**Figure 11:**
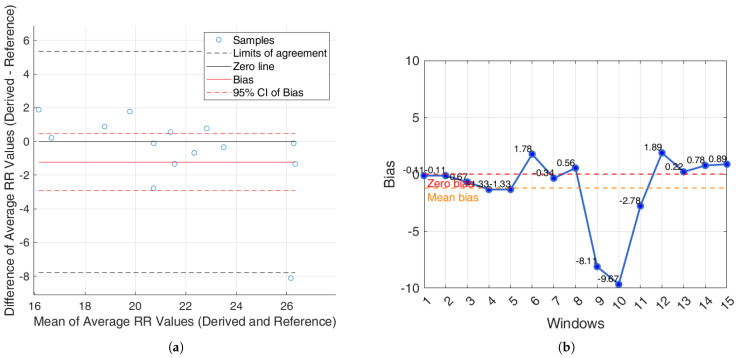
Capnobasedataset. (**a**) Bland–Altman plot for the R-peak based on inter-subject analysis, showing agreement between the average derived and reference RRs; (**b**) bias computed on each window (breaths/min). The red line indicates the zero-bias reference, while the orange line represents the mean bias.

For the Capnobase dataset, the BA analysis based on the agreement between subjects, which shows the average RR (derived and reference), is shown in Figure 11a. In Figure 11b, the bias for each window is reported.

## 4. Discussion

Numerous methods have been proposed in the literature for extracting EDR and PDR signals, aiming to enable automated and non-invasive monitoring of RR in both clinical and everyday settings. These techniques are mainly divided into two categories, namely, filter-based and feature-based approaches [19,25]. Filter-based methods apply band-pass filters directly to isolate respiratory frequency components [48,49], while feature-based approaches extract beat-by-beat characteristics (e.g., QRS area and duration, peak amplitude, PPG pulse width) that reflect respiratory modulation [30,50,51,52]. In 2016, Charlton et al. [25] conducted a comprehensive comparison of various algorithms for estimating RR from ECG and PPG signals, under both ideal and real clinical conditions. Their findings showed that algorithms generally performed better when applied to ECG rather than PPG signals.

The present work lies in the proposal of a methodological pipeline for RR estimation, based on the extraction of morphological features from ECG and PPG signals, aimed at identifying the most accurate and robust features. Although we focus on a single algorithm and a limited set of six features, the proposed method is simple to implement, computationally efficient, and relies on key fiducial points commonly used for both ECG and PPG. To validate the pipeline, two publicly available datasets, which include both healthy and pathological subjects, were used, allowing us to assess performance across different populations. Additionally, RR estimation was evaluated across all time windows of the signals, providing a realistic measure of performance across varying signal quality. In line with previous literature [36,37], results demonstrated that a 30-s time window yielded the highest accuracy for RR estimation. By contrast, although Charlton et al. [25] also used 32-s windows, they excluded segments with poor signal quality, meaning their reported performance is based only on clean, high-quality windows. Our approach, therefore, offers a more representative evaluation under real-world conditions.

Based on the obtained results, it is possible to observe that the derived signal and the reference signal oscillate with a similar pattern, especially in the case of ECG-derived signals, which allowed for the reconstruction of the EDR signal with good performance. In contrast, while the signal derived from the PPG also displayed a similar visual pattern, it exhibited a higher oscillatory rate. To further detail, we performed a quantitative evaluation by calculating error metrics in order to provide a more accurate assessment of each method (Table 1). Indeed, the ECG-features methods showed the lowest values compared to the PPG-features methods in both datasets. In accordance with previous findings reported in the literature [25], the findings of the present study suggest that the algorithms for RR estimation based on ECG features outperform those relying on PPG characteristics. The ECG features are intentionally simple and interpretable, mainly based on amplitudes and slopes around the R-wave. This simplicity offers advantages, especially in terms of computational load and physiological interpretability, thereby facilitating simpler real-time applications. While our study did not directly explore the physiological mechanisms underlying these features, previous research has linked QRS slopes to cardiac ischemia biomarkers and identified variations in R-wave amplitude as relevant in contexts such as sleep apnea detection [42]. These results are further supported by the statistical analyses performed. From the Wilcoxon signed rank test within the within-subject analysis, the derived breathing rates from the ECG-derived features are very close to the reference values, with few statistically significant differences (Table 2 and Table 3). A similar trend was observed in the between-subjects analysis (Table 4 and Table 5). In contrast, PPG-derived features exhibited a higher number of statistically significant differences, suggesting greater variability and reduced consistency in estimating RR both within and between subjects (Table 4 and Table 5).

Additionally, an agreement analysis was performed to evaluate the consistency between estimated and reference respiratory signals, offering a holistic view of each method’s reliability. ECG-derived features (R-peak, QRS area, up-slope, down-slope) exhibit relatively small bias values across both datasets and conditions, with most values close to or below ±1 breaths/min. Among the ECG-features, the R-peak showed the lowest error rates, confirming its superior reliability for RR estimation. In particular, the RR predicted by the proposed algorithms showed promising results, aligning well with the reference respiratory signals. The BA plots based on inter-subject agreement show a mean difference (bias) of −0.97 breaths/min in iAMwell and −1.16 breaths/min in Capnobase, indicating a systematic underestimation by the derived method. All data points fall within the limits of agreement, suggesting good consistency between the two methods. No apparent trend is observed between the differences and the mean values. Similarly, the intra-subject BA analysis reveals a bias of −0.84 breaths/min in iAMwell and −1.22 breaths/min in the Capnobase, again indicating systematic underestimation. In this case, all data points also lie within the agreement limits, further confirming the reliability of the derived method. In contrast, the PPG-derived features (FM and AM) show greater variability. While AM and FM components on the iAMwell dataset present moderately higher bias. In particular, the AM values remain relatively stable between intra- and inter-subject evaluations. Notably, the Capnobase dataset reveals more pronounced fluctuations—FM exhibits a high positive bias in both the intra- and inter-subject analyses (13.45 and 19.83 breaths/min, respectively). Additionally, the limits of agreement are wider in the inter-subject analysis. This may be attributed to the greater variability among subjects, as also suggested by Pimentel et al. [53]. Consistent with this trend, it is worth noting that ECG-based methods tend to underestimate the RR, whereas PPG-based methods generally overestimate it.

Although PPG is widely used in wearable devices due to its ease of acquisition and non-invasiveness, respiration signals derived from PPG showed lower performance compared to those obtained from ECG. This can be attributed to PPG’s greater sensitivity to motion artifacts, especially during physical activity, but also to vascular and tissue changes that may occur even in the absence of movement, such as in clinically ill patients. Factors like vasoconstriction, hypothermia, a deep gasp, or a yawn could degrade the signal and impact the accuracy of PPG-derived respiratory rate estimates [54,55,56]. Considering our results, the respiratory signal in the Capnobase dataset is of the capnographic type, meaning it is measured based on CO_2_ oscillations. This signal is sensitive to changes in pulmonary blood flow distribution and perfusion, and it may be less reliable than thoracic respiratory measurements. In contrast, the iAMwell dataset captures respiratory activity through variations in abdominal and thoracic circumference using inductive sensors. Considering that AM in the PPG signal is primarily driven by intrathoracic pressure changes [19] and not directly influenced by ventilation, we hypothesize that the PPG signal may correlate more strongly with the inductive respiratory signal from the iAMwell dataset, based on thoracic and abdominal circumferences, than with the capnogram from the Capnobase dataset, which reflects CO_2_ oscillations. This could explain the observed differences in performance between the two datasets.

Considering FM, as mentioned in the introduction, it originates from RSA, a physiological response in which respiration induces changes in intrathoracic pressure. For example, inhalation induces an increase that stretches the sinoatrial node and increases heart rate. Since ECG directly captures the heart’s electrical activity, it provides a more direct and accurate measure of RSA-induced FM compared to PPG.

Finally, it is worth noting that the findings from the iAMwell dataset showed that the acceleration phase of the exercise protocol revealed a clear increase in RR, consistent with the expected physiological response to exercise. Starting from a baseline of 19 breaths/min, RR gradually increased as treadmill speed accelerated, reaching up to 22 breaths/min. This trend reflects the body’s natural adjustment to physical exertion, confirming that the algorithm effectively captures the expected changes in breathing rate during exercise, making it a reliable tool for monitoring respiratory responses to varying intensities.

While only a limited number of studies have simultaneously leveraged both ECG and PPG signals [26,27,28,29,30], the majority of existing research has primarily focused on the technical development of filters and signal reconstruction methods [31,32,33], rather than on analyzing how different features extracted from source signals (i.e., ECG or PPG) impact the accuracy of RR estimation. As a result, direct comparison with existing literature is not straightforward.

A key strength of this study is the use of two complementary datasets, including both healthy and pathological subjects, which allows a more robust and clinically relevant evaluation of the proposed RR estimation methods. As a future development, working with more customized and clinically diverse datasets is still recommended, as this would allow for a more comprehensive validation and further optimization of the proposed approach. Future work will also explore the fusion of features from both ECG and PPG signals to improve estimation accuracy. Moreover, the influence of various technical (e.g., sampling frequency, measurement site, protocol), clinical (e.g., diseases, comorbidities), and socio-demographic (e.g., age, gender, body max index) factors on signal quality and reliability will be thoroughly investigated. Such an approach could ultimately offer a low-cost and efficient tool for early screening of potential pathologies or serve as a set of biomarkers for unhealthy lifestyle detection.

## 5. Conclusions

In conclusion, this study demonstrated the possibility of extracting respiratory patterns from both ECG and PPG signals. However, quantitative analysis showed that the signals derived from the ECG yielded more accurate results compared to PPG. The analysis highlighted that ECG-derived features provide more reliable estimations compared to PPG-based ones. The proposed feature selection approach allowed for the identification of the most informative parameters, improving robustness against intra- and inter-subject variability. These findings represent a promising step toward the development of cheap, non-invasive, and easily deployable respiration monitoring systems. Potential clinical applications may include continuous respiratory monitoring in intensive care units, post-operative settings, or during anesthesia, as well as remote and home-based monitoring for patients with chronic respiratory diseases such as asthma or sleep apnea. Future research will further validate and extend these results to broader and more diverse populations and conditions.

## Figures and Tables

**Table 1:** Performance metrics of ECG morphological characteristics and PPG with AM and FM techniques.

	R-Peak	QRS Area	Up-Slope	Down-Slope	FM	AM
**iAMwell dataset**						
**MAE**	0.99	1.02	1.02	1.04	5.10	5.12
**MAPE (%)**	9.45	9.78	9.64	9.94	26.65	27.17
**RMSE**	2.75	2.86	2.83	2.68	6.10	6.11
ρ	0.700 *	0.684 *	0.672 *	0.600 *	<0.5	<0.5
**Capnobase dataset**						
**MAE**	3.07	3.74	3.19	3.32	13.90	10.66
**MAPE (%)**	27.33	36.50	29.33	29.95	136.77	105.65
**RMSE**	11.71	13.46	13.41	12.90	181.66	181.66
ρ	0.642 *	0.535 *	0.497 *	0.476 *	<0.5	<0.5

ECG = electrocardiogram; PPG = photoplethysmogram; AM = amplitude modulation; FM = frequency modulation; MAE = mean absolute error; MAPE = mean absolute percentage error; RMSE = root mean square error; ρ = Pearson correlation coefficient. * Statistically significant with *p* < 0.05.

**Table 2:** Intra-subject estimates for the iAMwell dataset. Comparison of estimated and reference RR within-subject. For each subject, the mean ± standard deviation of RR (breaths/min) estimates is reported along with the corresponding *p*-values assessing temporal variation.

	Reference	R-Peak		QRS Area		Up-Slope		Down-Slope		FM		AM	
Patient	Mean ± SD	Mean ± SD	*p*-Value	Mean ± SD	*p*-Value	Mean ± SD	*p*-Value	Mean ± SD	*p*-Value	Mean ± SD	*p*-Value	Mean ± SD	*p*-Value
Subject 1	21.47±3.07	21.33±4.25	0.719	20.80±4.77	0.093	20.93±4.46	0.087	21.33±4.32	0.698	21.20±3.98	0.703	23.46±3.51	0.105
Subject 2	24.27±2.37	23.73±1.98	0.366	23.60±2.03	0.272	23.73±2.25	0.305	22.87±2.25	0.417	20.53±3.58	0.006 *	22.67±2.89	0.145
Subject 3	20.93±3.70	18.93±3.69	0.011 *	18.93±3.69	0.011 *	18.93±3.69	0.011 *	19.07±3.37	0.008 *	20.80±4.87	0.850	22.53±4.30	0.257
Subject 4	17.73±1.98	17.47±2.44	0.546	16.93±2.37	0.083	17.20±2.24	0.206	17.73±1.98	0.454	22.00±4.84	0.018 *	23.87±5.73	0.004 *
Subject 5	18.40±2.03	18.27±2.82	0.774	18.13±2.88	0.527	18.00±2.93	0.408	18.40±2.03	0.317	20.53±5.63	0.205	22.40±5.51	0.019 *
Subject 6	18.40±3.04	16.93±3.11	0.041 *	17.07±3.01	0.150	17.20±3.45	0.098	16.93±2.49	0.027 *	30.93±4.20	0.062	23.87±5.26	0.007 *
Subject 7	22.00±2.73	20.80±2.58	0.146	20.53±2.56	0.098	20.13±2.33	0.045 *	19.60±2.41	0.011 *	18.40±3.79	0.011 *	22.13±4.86	0.937
Subject 8	18.67±2.69	16.53±1.41	0.007 *	16.67±1.80	0.011 *	16.27±2.49	0.018 *	16.27±2.49	0.014 *	20.40±5.13	0.194	22.80±5.64	0.022 *
Subject 9	20.53±2.67	18.53±2.77	0.497	18.13±.20	0.398	18.67±2.23	0.485	17.87±2.45	0.365	20.26±3.85	0.937	22.53±4.87	0.138
Subject 10	19.67±1.84	20.00±1.85	0.882	19.33±1.45	0.719	19.33±2.09	0.756	19.20±1.90	0.426	21.61±4.22	0.081	23.87±4.81	0.025 *
Subject 11	19.73±3.11	19.32±2.89	0.726	18.93±2.91	0.654	19.33±2.99	0.687	18.93±2.25	0.654	25.73±3.01	0.002 *	22.67±3.59	0.045 *
Subject 12	22.27±4.77	21.07±2.92	0.446	20.13±2.77	0.055	19.87±2.45	0.070	20.67±3.35	0.062	26.13±3.33	0.031 *	24.40±3.13	0.063
Subject 13	21.73±2.60	19.87±2.97	0.029 *	20.40±3.14	0.093	20.27±3.82	0.070	20.27±3.20	0.084	26.41±3.14	0.004 *	26.27±3.79	0.008 *
Subject 14	18.00±3.12	18.00±3.55	0.951	17.87±3.42	0.856	18.13±4.10	0.884	18.27±3.28	0.786	26.93±3.69	0.001 *	25.73±3.53	0.001 *
Subject 15	21.87±3.42	22.40±3.20	0.671	22.62±3.40	0.595	22.77±3.00	0.399	22.97±3.01	0.476	25.86±3.50	0.043 *	26.13±3.96	0.040 *

Mean ± SD = media ± standard deviation; * Statistical significance at 0.05.

**Table 3:** Intra-subject estimates for the Capnobase dataset. Comparison of estimated and reference RR within-subject. For each subject, the mean ± standard deviation of RR (breaths/min) estimates is reported along with the corresponding *p*-values assessing temporal variation.

	Reference	R-Peak		QRS Area		Up-Slope		Down-Slope		FM		AM	
Patient	Mean ± SD	Mean ± SD	* p *	Mean ± SD	* p *	Mean ± SD	* p *	Mean ± SD	* p *	Mean ± SD	* p *	Mean ± SD	* p *
Subject 1	20.53 ± 5.15	20.93 ± 3.45	0.497	22.13 ± 3.16	0.165	21.20 ± 3.45	0.472	21.73 ± 3.01	0.170	32.67 ± 12.25	0.001 *	27.20 ± 12.35	0.068
Subject 2	21.07 ± 5.28	21.33 ± 3.52	0.611	22.80 ± 2.24	0.206	21.07 ± 3.20	0.651	20.93 ± 3.69	0.887	32.13 ± 11.45	0.001 *	28.13 ± 9.87	0.005 *
Subject 3	19.87 ± 4.44	20.40 ± 3.56	0.595	23.07 ± 3.20	0.016 *	20.93 ± 3.53	0.253	20.67 ± 3.44	0.436	35.20 ± 15.06	0.001 *	29.73 ± 13.67	0.005 *
Subject 4	21.73 ± 4.56	19.73 ± 3.99	0.178	21.60 ± 3.87	0.655	20.00 ± 4.96	0.383	19.73 ± 4.83	0.265	35.47 ± 14.55	0.003 *	30.53 ± 13.64	0.038 *
Subject 5	22.53 ± 6.29	20.80 ± 4.39	0.085	21.60 ± 3.87	0.726	22.93 ± 3.10	0.449	21.47 ± 4.03	0.445	37.33 ± 14.90	0.001 *	30.40 ± 14.27	0.027 *
Subject 6	22.40 ± 6.21	20.67 ± 4.76	0.255	22.27 ± 3.45	0.975	20.80 ± 4.39	0.384	20.53 ± 4.44	0.218	34.53 ± 13.32	0.002 *	28.93 ± 12.96	0.059
Subject 7	22.53 ± 5.14	21.73 ± 4.53	0.719	23.73 ± 3.01	0.277	21.73 ± 4.33	0.277	21.60 ± 3.94	0.691	37.47 ± 15.01	0.001 *	31.07 ± 14.08	0.032 *
Subject 8	23.60 ± 6.02	22.67 ± 4.05	0.437	22.80 ± 3.10	0.472	22.40 ± 4.42	0.201	22.00 ± 4.00	0.136	37.07 ± 15.75	0.002 *	31.87 ± 15.11	0.047 *
Subject 9	23.47 ± 5.80	21.87 ± 4.69	0.400	22.67 ± 3.90	0.636	22.00 ± 4.66	0.371	22.27 ± 4.46	0.474	35.20 ± 12.35	0.003 *	31.07 ± 13.41	0.042 *
Subject 10	20.93 ± 5.48	21.33 ± 2.89	0.591	21.87 ± 2.77	0.245	21.20 ± 2.91	0.797	20.93 ± 3.01	0.786	33.47 ± 12.11	0.001 *	27.60 ± 11.44	0.043 *
Subject 11	24.13 ± 6.01	21.47 ± 4.44	0.070	21.60 ± 3.14	0.065	20.80 ± 4.83	0.035 *	21.33 ± 4.51	0.096	36.27 ± 13.22	0.003 *	32.53 ± 14.86	0.049 *
Subject 12	23.60 ± 5.18	20.13 ± 5.26	0.011 *	20.80 ± 3.76	0.124	21.07 ± 4.95	0.022 *	20.53 ± 5.32	0.036 *	36.27 ± 15.10	0.005 *	32.27 ± 15.91	0.042 *
Subject 13	21.73 ± 5.74	21.07 ± 4.27	0.821	22.67 ± 2.35	0.410	21.33 ± 4.45	0.753	20.80 ± 4.33	0.550	37.73 ± 15.13	0.001 *	33.33 ± 15.49	0.014 *
Subject 14	22.67 ± 5.68	20.93 ± 4.53	0.410	22.13 ± 3.34	0.892	21.20 ± 4.13	0.321	20.93 ± 4.46	0.372	36.80 ± 13.75	0.002 *	33.33 ± 15.32	0.022 *
Subject 15	22.53 ± 6.15	20.80 ± 3.36	0.410	21.87 ± 2.77	0.423	21.07 ± 3.28	0.290	20.40 ± 2.85	0.124	37.47 ± 12.59	0.001 *	33.20 ± 13.39	0.011 *
Subject 16	22.40 ± 5.01	20.53 ± 4.50	0.570	22.53 ± 3.50	0.419	21.47 ± 4.87	0.720	20.27 ± 4.13	0.085	35.60 ± 13.65	0.001 *	30.93 ± 15.36	0.044 *
Subject 17	22.00 ± 5.01	21.07 ± 5.18	0.557	22.13 ± 3.25	0.419	21.87 ± 4.31	0.720	21.33 ± 4.19	0.085	37.60 ± 13.42	0.001 *	30.40 ± 14.99	0.019 *
Subject 18	22.40 ± 5.72	20.67 ± 4.39	0.095	21.73 ± 3.77	0.771	20.93 ± 4.65	0.133	21.07 ± 4.13	0.347	35.33 ± 15.71	0.004 *	30.27 ± 15.80	0.043 *
Subject 19	21.75 ± 5.21	22.20 ± 4.75	0.078	21.73 ± 3.77	0.771	20.93 ± 4.65	0.133	21.07 ± 4.13	0.347	35.33 ± 15.71	0.004 *	30.27 ± 15.80	0.043 *

Mean ± SD = media ± standard deviation; * Statistical significance at 0.05.

**Table 4:** Inter-subject estimates for the iAMwell dataset. Comparison of estimated and reference RR between subjects. For each subject, the mean ± standard deviation of RR (breaths/min) estimates is reported along with the corresponding *p*-values assessing temporal variation.

	Reference	R-Peak		QRS Area		Up-Slope		Down-Slope		FM		AM	
Window	Mean ± SD	Mean ± SD	*p*-Value	Mean ± SD	*p*-Value	Mean ± SD	*p*-Value	Mean ± SD	*p*-Value	Mean ± SD	*p*-Value	Mean ± SD	*p*-Value
Window 1	20.00±2.94	19.00±3.40	0.614	19.43±3.27	0.350	19.29±3.38	0.185	19.29±3.73	0.233	25.20±3.61	0.002 *	27.73±2.72	0.001 *
Window 2	19.60±3.22	19.70±3.40	0.415	19.07±3.28	0.635	18.67±2.89	0.654	19.20±3.36	0.521	23.87±2.97	0.467	22.40±3.79	0.099
Window 3	19.73±3.01	18.67±3.60	0.132	18.67±3.60	0.043 *	18.13±3.89	0.046 *	18.53±3.58	0.021 *	24.13±6.02	0.023 *	24.67±3.83	0.007 *
Window 4	19.47±3.07	18.27±3.20	0.754	17.87±3.25	0.107	18.27±3.20	0.010 *	17.73±2.81	0.010 *	20.93±5.55	0.046 *	20.13±4.03	0.022 *
Window 5	19.20±2.24	18.67±3.35	0.771	18.00±3.21	0.047 *	17.87±2.67	0.026 *	18.53±3.60	0.340	19.87±4.44	0.160	20.53±3.81	0.026 *
Window 6	19.57±3.16	18.86±2.57	0.238	18.71±2.55	0.230	19.14±2.57	0.546	18.43±2.50	0.201	28.00±3.12	0.001 *	31.33±4.11	0.001 *
Window 7	19.71±2.58	19.60±4.22	0.218	19.60±4.29	0.301	19.33±4.25	0.365	19.60±3.94	0.342	23.60±2.64	0.164	24.67±3.97	0.069
Window 8	20.27±3.61	18.80±3.61	0.088	18.93±3.37	0.102	18.80±3.69	0.093	18.67±3.35	0.078	20.27±2.60	0.111	22.40±2.64	0.007 *
Window 9	19.43±3.96	18.40±3.48	0.465	18.43±3.34	0.421	18.40±3.64	0.463	18.13±3.50	0.078	22.93±5.75	0.089	23.87±3.74	0.005 *
Window 10	19.47±4.10	18.67±3.90	0.881	18.00±3.81	0.077	18.13±3.58	0.089	18.23±3.81	0.075	20.67±4.93	0.369	21.33±3.67	0.086
Window 11	19.60±3.31	19.47±3.66	0.473	19.47±3.16	0.475	19.20±3.10	0.387	19.07±3.01	0.286	23.33±2.46	0.004 *	25.07±2.49	0.002 *
Window 12	21.73±3.01	20.40±3.22	0.235	20.40±3.04	0.201	20.13±3.50	0.352	19.87±3.16	0.038 *	20.93±5.16	0.164	24.00±3.21	0.025 *
Window 13	22.27±3.61	21.07±1.98	0.216	21.33±2.96	0.281	21.07±2.71	0.213	21.33±2.99	0.212	19.33±6.35	0.043 *	20.40±5.19	0.025 *
Window 14	22.80±3.99	21.60±3.56	0.546	20.80±3.61	0.045 *	21.45±3.74	0.410	21.47±3.66	0.421	24.00±2.82	0.042 *	24.27±2.91	0.039 *
Window 15	22.80±3.99	22.00±2.73	0.498	21.60±2.53	0.081	21.47±3.16	0.075	21.60±2.53	0.082	20.67±4.26	0.042 *	22.53±2.97	0.687

Mean ± SD = media ± standard deviation; * Statistical significance at 0.05.

**Table 5:** Inter-subject estimates for the Capnobase dataset. Comparison of estimated and reference RR between subjects. For each subject, the mean ± standard deviation of RR (breaths/min) estimates is reported along with the corresponding *p*-values assessing temporal variation.

	Reference	R-Peak		QRS Area		Up-Slope		Down-Slope		FM		AM	
Window	Mean ± SD	Mean ± SD	*p*-Value	Mean ± SD	*p*-Value	Mean ± SD	*p*-Value	Mean ± SD	*p*-Value	Mean ± SD	*p*-Value	Mean ± SD	*p*-Value
Window 1	20.78 ± 3.51	20.67 ± 4.00	0.655	22.89 ± 2.59	0.003 *	20.67 ± 4.00	0.655	20.56 ± 3.68	0.317	34.56 ± 4.16	0.001 *	27.11 ± 3.23	0.001
Window 2	26.33 ± 2.85	26.22 ± 2.73	0.705	26.22 ± 3.06	0.803	26.33 ± 2.59	0.987	26.44 ± 2.96	0.739	39.22 ± 5.28	0.001 *	23.11 ± 3.01	0.001 *
Window 3	22.67 ± 1.81	22.00 ± 1.68	0.163	21.89 ± 1.75	0.216	21.89 ± 2.11	0.218	22.11 ± 2.11	0.449	26.67 ± 3.22	0.001 *	21.89 ± 4.42	0.398
Window 4	22.22 ± 1.93	20.89 ± 2.19	0.017 *	21.56 ± 2.43	0.442	20.78 ± 2.49	0.035 *	21.22 ± 2.49	0.136	27.67 ± 3.16	0.001 *	27.11 ± 3.08	0.001 *
Window 5	27.00 ± 4.41	25.67 ± 5.19	0.158	25.00 ± 5.14	0.049 *	25.56 ± 5.11	0.086	24.33 ± 5.58	0.010 *	42.89 ± 7.80	0.001 *	35.33 ± 4.12	0.001
Window 6	18.89 ± 3.89	20.67 ± 2.57	0.109	22.44 ± 1.89	0.010 *	21.22 ± 2.84	0.037 *	21.11 ± 2.49	0.023 *	22.56 ± 3.20	0.008 *	22.67 ± 2.91	0.003 *
Window 7	23.67 ± 2.50	23.33 ± 2.17	0.716	23.11 ± 1.41	0.272	23.89 ± 1.45	0.762	22.33 ± 1.97	0.051	29.44 ± 7.94	0.012 *	22.11 ± 3.66	0.138
Window 8	21.11 ± 3.16	21.67 ± 2.93	0.132	22.11 ± 3.18	0.020 *	21.67 ± 3.16	0.132	21.56 ± 3.11	0.248	32.78 ± 7.23	0.001 *	21.00 ± 3.45	0.808
Window 9	30.22 ± 7.29	22.11 ± 3.32	0.002 *	21.67 ± 2.50	0.001 *	22.44 ± 3.18	0.001 *	21.56 ± 1.76	0.001 *	37.22 ± 5.66	0.008 *	30.89 ± 5.95	0.532
Window 10	25.89 ± 3.85	16.22 ± 3.28	0.001 *	20.56 ± 3.20	0.002 *	15.89 ± 2.95	0.001 *	16.11 ± 3.60	0.001 *	54.00 ± 10.74	0.001 *	57.89 ± 10.21	0.001 *
Window 11	22.11 ± 4.36	19.33 ± 2.99	0.019 *	20.11 ± 2.32	0.071	20.00 ± 2.91	0.028 *	19.44 ± 2.81	0.010 *	42.00 ± 12.14	0.001 *	39.00 ± 13.32	0.001 *
Window 12	15.22 ± 1.22	17.11 ± 2.30	0.013 *	21.11 ± 2.76	0.001 *	18.44 ± 2.96	0.003 *	18.44 ± 2.53	0.002 *	22.11 ± 2.11	0.001 *	16.11 ± 5.16	0.473
Window 13	16.56 ± 2.97	16.78 ± 1.70	0.633	20.00 ± 2.47	0.009 *	17.11 ± 2.19	0.459	17.22 ± 2.49	0.403	40.11 ± 20.17	0.001 *	39.00 ± 16.88	0.001 *
Window 14	22.44 ± 5.47	23.22 ± 4.56	0.357	23.78 ± 2.73	0.172	22.89 ± 4.40	0.475	22.44 ± 5.25	0.795	35.44 ± 16.66	0.049 *	32.78 ± 14.18	0.403
Window 15	18.33 ± 2.93	19.22 ± 2.49	0.351	22.00 ± 2.83	0.005	20.11 ± 3.03	0.113	20.56 ± 2.64	0.041	49.67 ± 19.96	0.001 *	44.67 ± 16.12	0.001 *

Mean ± SD = media ± standard deviation; * Statistical significance at 0.05.

**Table 6:** Bland–Altman analysis: bias and limit of agreement (in breaths/min) for each method across the two datasets.

Method	R-Peak	QRS Area	Up-Slope	Down-Slope	FM	AM
**iAMwell (Intra-subject)**						
Bias	−0.97	−0.95	−1.02	−1.07	2.49	3.66
Lower Limit	−2.62	−2.76	−2.91	−3.21	−5.71	−1.34
Upper Limit	0.95	0.68	0.92	1.17	11.32	7.69
**Capnobase (Intra-subject)**						
Bias	−1.16	0.11	−1.00	−1.16	13.45	8.52
Lower Limit	−3.37	−2.80	−3.07	−3.36	10.72	5.74
Upper Limit	1.10	2.79	1.28	1.01	16.34	11.23
**iAMwell (Inter-subject)**						
Bias	−0.84	−0.95	−1.02	−1.07	2.74	3.31
Lower Limit	−1.76	−2.05	−1.92	−2.05	−3.78	−3.34
Upper Limit	0.20	0.10	−0.25	0.10	8.06	9.96
**Capnobase (Inter-subject)**						
Bias	−1.22	0.07	−0.97	−1.20	19.83	8.48
Lower Limit	−7.91	−7.17	−7.91	−8.37	−4.35	−13.10
Upper Limit	5.35	7.30	5.97	5.97	30.75	29.87

## Data Availability

The data used in this study are publicly available as described in [34,35].

## Abbreviations

The following abbreviations are used in this manuscript:

AM	Amplitude Modulation
BA	Bland-Altman
ECG	Electrocardiogram
EDR	Electrocardiogram-Derived Respiration
FM	Frequency Modulation
MAE	Mean Absolute Error
MAPE	Mean Absolute Percentage Error
PDR	Photoplethysmography Derived Respiration
PPG	Photoplethysmography
RMSE	Root Mean Square Error
RR	Respiratory Rate
RSA	Respiratory Sinus Arrhythmia
SD	Standard Deviation
